# Transformation of Selected Trichothecenes during the Wheat Malting Production

**DOI:** 10.3390/toxins13020135

**Published:** 2021-02-11

**Authors:** Marcin Bryła, Edyta Ksieniewicz-Woźniak, Dorota Michałowska, Agnieszka Waśkiewicz, Tomoya Yoshinari, Romuald Gwiazdowski

**Affiliations:** 1 Department of Food Safety and Chemical Analysis, Prof. Waclaw Dabrowski Institute of Agricultural and Food Biotechnology, 02-532 Warsaw, Poland; edyta.wozniak@ibprs.pl; 2 Beer and Malt Laboratory, Prof. Waclaw Dabrowski Institute of Agricultural and Food Biotechnology, 02-532 Warsaw, Poland; dorota.michalowska@ibprs.pl; 3 Department of Chemistry, Faculty of Forestry and Wood, Poznan University of Life Sciences, 60-625 Poznan, Poland; agnieszka.waskiewicz@up.poznan.pl; 4 Division of Microbiology, National Institute of Health Sciences, Kawasaki-ku, Kawasaki-shi, Kanagawa 210-9501, Japan; t-yoshinari@nihs.go.jp; 5 Research Centre for Registration of Agrochemicals, Institute of Plant Protection–National Research Institute, 60-318 Poznań, Poland; R.Gwiazdowski@iorpib.poznan.pl

**Keywords:** wheat, malting, deoxynivalenol, nivalenol, biotransformation, modified mycotoxins

## Abstract

The transformation of deoxynivalenol (DON), nivalenol (NIV), and their glucosides (DON3G and NIV3G) during the malting of grains of two wheat varieties was studied. The concentration of DON3G and NIV3G started to increase significantly before the concentration of DON and NIV increased. This may reflect the transformation of the parent mycotoxin forms into their glucosides due to xenobiotic detoxification reactions. After a sharp rise during the last 2 days of the process (day 6 and 7), the DON concentration reached 3010 ± 338 µg/kg in the Legenda wheat-based malt and 4678 ± 963 µg/kg in the Pokusa wheat-based malt. The NIV concentration, at 691 ± 65 µg/kg, remained the same as that in the dry grain. The concentration of DON3G in the Legenda and Pokusa wheat-based malt was five and three times higher, respectively, than that in the steeped grain. The concentration of NIV3G in the Legenda wheat-based malt was more than twice as high as that in the steeped grain. The sharp increase in the concentration of DON at the end of the malting process reflected the high pathogen activity. We set aside some samples to study a batch that was left undisturbed without turning and aeration, for the entire period of malting. The concentration of DON in the malt produced from the latter batch was 135% and 337% higher, for Legenda and Pokusa, respectively, than that in the malt produced from the batch that was turned and aerated. The NIV concentration was 22% higher in the latter batch.

## 1. Introduction

Wheat (*Triticum aestivum*) is among the main food crops cultivated worldwide. It is considered highly nutritious and contains minerals, vitamins, and lipids [1]. Hence, it is widely used in various cuisines as a popular ingredient. It is also used as a raw material for the production of malt and beer [2]. High quality cereals are required to obtain good quality malt [3,4].

However, during the growing season, wheat can become infected with fungi, mostly of the *Fusarium* genus [5]. *Fusarium graminearum* and *F. culmorum* are the fungal species that are dominant in wheat. The fungi grow optimally at temperatures between 20 and 25 °C and air humidity above 80% [6]. Their variability and frequency of occurrence depends mainly on climatic changes over a period of years and on agricultural practices [7,8]. The susceptibility of various plants to infection depends on the fungal species, plant variety, level of nutrients in the soil, and presence of pests and other microorganisms [6]. Proper agrotechnical practices such as crop rotation, use of fungicides, and selection of cereal varieties with lower susceptibility to the fungi may partially inhibit the development of *Fusarium* head blight (FHB). FHB, a typical disease of cereals, causes significant losses by reducing the crop quality and volume of the harvested grain [9,10]. Fungal secondary metabolites are toxic, and the main mycotoxins include a range of fumonisins and trichothecenes. Deoxynivalenol (DON) and nivalenol (NIV) are the most prevalent trichothecenes (B group). Their modified forms are also found often in malting and brewing products [3,10,11]. DON toxicity has been studied thoroughly in humans and animals, and the most frequently observed symptoms include chronic and/or acute disorders of the gastrointestinal tract such as impaired nutrient absorption, intestinal inflammation, and disruption of the local intestinal immune response [12,13,14]. At the subcellular level, DON inhibits the synthesis of proteins, nucleic acids [15], and DNA; reduces cell proliferation; and leads to cell apoptosis [16,17]. NIV has similar toxic effects. However, there is lesser information on NIV as compared to that on other trichothecenes, in the literature. Some studies have indicated the synergistic effects of various trichothecenes due to their natural co-occurrence in cereals [18]. Mycotoxins not only jeopardise food safety but also degrade the quality of malt and beer [4].

Deoxynivalenol-3-glucoside (DON3G) and nivalenol-3-glucoside (NIV3G) are modified forms of DON and NIV, respectively. DON3G and NIV3G may be the products of metabolic reactions in infected plants or may be produced directly by the fungi [19,20]. Lately, a lot of research is being carried out on these modified forms. Similar to their parent forms, they may occur naturally in cereals and may affect food quality and safety. The DON3G concentration in malt may sometimes even be higher than the concentration of DON, although the published reports are inconclusive [21,22]. To date, NIV3G has not been found in malt.

The European Commission has stipulated that the acceptable concentration of DON in unprocessed cereals (except durum wheat, oats, and maize) should not exceed 1250 µg/kg [23]. However, no regulations have been established for the acceptable concentration of DON in barley and wheat malt, for the acceptable concentration of NIV in any foodstuff, or for the acceptable concentrations of the modified forms of DON and NIV in any foodstuff.

Nevertheless, in 2017, the European Food Safety Authority (EFSA) issued a scientific opinion on the risks to humans and animals caused by the presence of DON and its acetylated/modified forms. As in case of the acetylated derivatives, the toxicity of the DON3G derivative is considered to be equal to that of DON, as DON3G has the potential to be hydrolyzed to free form DON in the gastrointestinal tract. Based on the collected epidemiological data, the group reference dose (RfD) for these compounds at the level of 8 µg per kg of body weight was established [24]. Similarly, in relation to the NIV, considering the potential risk of NIV3G hydrolysis in the human gastrointestinal tract and its co-occurrence with NIV in cereal grains, the EFSA concluded that NIV3G should also be included in the group Tolerable Daily Intake (TDI) and RfD together with NIV. Unfortunately, the scientific data on the presence of DON3G, and especially NIV3G in food, is still limited [25].

The aim of this study was to investigate changes in the concentrations of DON, NIV, and their modified forms, DON3G and NIV3G, during the malting of two wheat cultivars while considering the pre-malting process of steeping the grain. The study also included an analysis of levels of the parent mycotoxin forms and their glucosides in wheat-based malt.

## 2. Results and Discussion

### 2.1. Regularly Turned/Aerated Malting Batch

DON and DON3G concentrations were 1540 ± 332 µg/kg and 236 ± 12 µg/kg, respectively, in the Legenda variety dry grain; and 2164 ± 210 µg/kg and 584 ± 143 µg/kg, respectively, in the Pokusa variety dry grain. NIV and NIV3G were found only in the Legenda variety dry grain, at a concentration of 702 ± 64 µg/kg and 239 ± 45 µg/kg respectively (see Table 1). These relatively high levels reflected the fact that the tested wheat was artificially inoculated with the *F. culmorum* pathogen. The intensity and outcome of natural infections (including mycotoxin levels in grain) depend mainly on weather conditions (rainfall and temperature) during the plant earing and flowering stages [26,27,28].

Conditions in which wheat grain is malted (temperature, relative humidity, access of fresh air) are favourable for secondary growth of microorganisms. It has been reported that the population of some microorganisms (including *Fusarium*) may significantly grow in the course of the wheat grain steeping and germination processes [29]. However, at the very beginning of the steeping, mycotoxins may also be rinsed off the grain, depending on their solubility in water. Literature data on decrease of mycotoxin levels after grain steeping are limited. For example, the reported reductions of DON in barley are: by 66% after 5 h of steeping and 19 h of air rest [8]; by 50–70% after similar treatment [30]; by 22% or 65% depending on the grain contamination level after 13 h of steeping and 4 h of air rest [31]; and down below LOD [32]. The steeping operations give rise predominantly to removal of mycotoxins adsorbed at the surface of the grains or mycotoxins contained in *Fusarium*-damaged grains that float on the water surface [33]. We noted some decrease of DON concentration after the grain steeping stage (decrease by 36%/24% for the Legenda/Pokusa wheat variety, respectively) (see Table 2), but both changes were statistically insignificant. On the other hand, the drop in NIV concentration (by 26%) was statistically significant. Our results were normalized to dry mass, hence reductions in mycotoxin concentrations may be relatively low as compared to the literature data. DON3G and NIV3G concentration did not change significantly. A possible explanation was offered by Freire and Sant’Ana [20]: Metabolites of endogenic reactions aimed to detoxicate basic forms of mycotoxins are stored in plant vacuoles and/or cell walls, hence the modified forms are more resistant to rinsing-off. On the other hand, the reactions may not be excluded even at this early processing stage (in the just germinating grain), which might increase the concentration of the modified forms. Our results on change of DON3G concentration during wheat steeping are in line with those concerning DON3G in steeped barley grain reported by Pascari et al. [31].

DON concentration stabilized in both tested wheat varieties between the grain steeping stage and day 4 of the malting period. Most probably that time was a period in which basic functions of the pathogens were stimulated. Between day 4 and 7, the concentration was steadily growing to reach finally about three times higher level than at the process beginning (the grain steeping stage): up to 3010 ± 338 µg/kg in the Legenda variety, and up to 4678 ± 963 µg/kg in the Pokusa, see Table 1. NIV concentration was slightly dropping until day 5 of the period, moderately increased on day 6, and clearly rose on the last day 7 of the period, up to 691 ± 65 µg/kg. The final/initial NIV concentration ratio was only 135% (while DON’s one was about 300%) (see Table 2). Perhaps the tested *Fusarium* fungi biosynthesize DON more efficiently than NIV. A visible growth of mycelium in the malted wheat grain accompanied the increase of concentration. Available literature data concern predominantly DON and are only partially in line with our results. Schwarz et al. [32] reported 18–114% increase of DON concentration during the barley grain malting process. On the other hand, Habler et al. [30] reported DON concentration increases as high as 300–700%.

**Figure 1:**
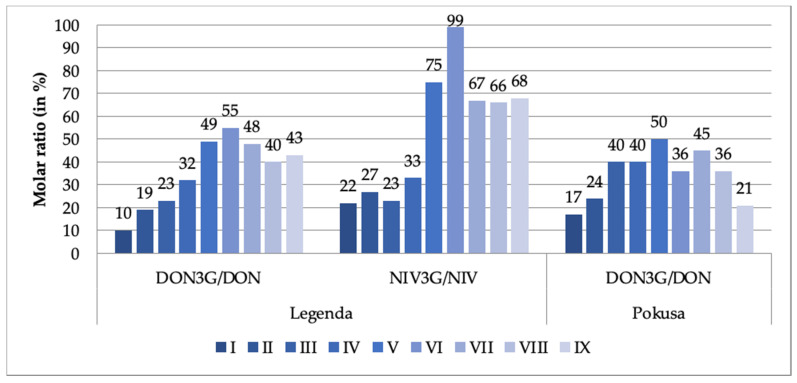
DON3G/DON and NIV3G/NIV molar ratios (%) at different stages of the wheat grain malting process (DON3G—deoxynivalenol-3-glucoside; DON—deoxynivalenol; NIV-3G—nivalenol-3-glucoside; NIV—nivalenol). The stage designations are provided in Table 1.

The observed dynamics were somewhat different in the case of the DON3G/NIV3G modified forms. Their concentrations did not significantly change until day 3, 4 or 5 of the malting period (depending on the mycotoxin and the wheat variety), then clearly increased. The final-to-initial (i.e., day 7 of the malting period-to-the just steeped grain) DON3G concentration ratio exceeded 500% in the Legenda variety and 300% in the Pokusa variety. NIV3G concentration grew somewhat less, by about 200%. Concentration of the glucosides started to grow earlier than DON/NIV concentration. It may be imagined that the grow reflected transformation of DON/NIV via the detoxification mechanisms put at work by the germinating grain, while steep rise in DON/NIV concentration (especially on day 6 and 7 of the malting period) reflected secondary biosynthesis of the mycotoxins by the pathogens. It is well known that increased production of mycotoxins is a typical reaction of fungi to a stress (e.g., lack of water or temperature fluctuations). Such a situation could have taken place as water content in the malted grain may have dropped from 45% down to 38% (dry mass analysis data). Glucosyltransferases are enzymes responsible for detoxification reactions that give rise to DON3G and NIV3G derivatives (among others). DON3G/DON ratios in both tested wheat varieties and NIV3G/NIV ratios in the Pokusa wheat variety were estimated in this work for each day of the grain malting process. The ratios are shown in Figure 1.

The DON3G/DON ratios in our dry grain samples were 10% (Legenda) and 17% (Pokusa). The range of ratios reported in the literature is 10–30% [22,34,35]. The ratio did not drop after steeping but was steadily increasing until day 4 or 5 (maximum 50–55%), and then was fluctuating (with a decreasing trend) until day 7 (see Figure 1). These results are only partly consistent with these reported by Habler et al. [30]; after steeping, they observed 2–2.5-fold increase in DON3G concentration as compared to dry grain, while the DON3G/DON ratio in the final malt exceeded 100%. We noted similar trends for NIV3G and NIV. The ratio was 22% in the Legenda variety dry grain samples and was growing (a bit less regularly than in case of the DON3G/DON ratio) until day 5 up to 99%. The fact that the amount of mycotoxin derivatives was rising reflects biochemical reactions running during the malting process. Literature concerning dynamics of these processes is sparse. The following ratios have been reported: 22–186% for DON3G/DON and 32–126% for NIV3G/NIV in barley malt [36]; and 45–82% for DON3G/DON in barley malt (we have recalculated the data published by Habschied et al. [37]). Bryła et al. [19], Ksieniewicz-Woźniak et al. [36], and Lancova et al. [38] report that both DON3G/DON and NIV3G/NIV ratios found in numerus tested samples of beer were also higher than 100%. Such data indicate a high activity of glucosyltransferases during the grain malting process.

### 2.2. Unturned Malting Batch

**Figure 2:**
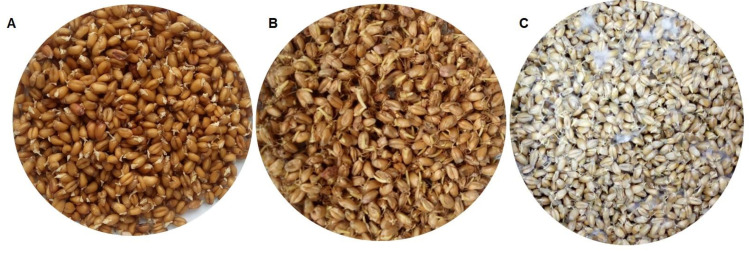
Malted wheat grain (Legend variety): (**A**) wheat grain after steeping; (**B**) malt after 7 days of malting (turned); (**C**) malt after 7 days of malting (unturned).

Regular turning of the malting batch is a basic operation in the malting industry performed to maintain a proper environment for correct development of germs and rootlets. The final malt produced from unturned batch contained more mycotoxins than the malt produced from a turned/aerated batch: by 135–337% more DON (depending on the wheat variety), by 157–156% more DON3G, by 22% more NIV, and by 25% more NIV3G (see, Table 2). We have found no literature data on the dependence of mycotoxin levels on batch turning and aeration. Visual inspection of the unturned batch confirms that much higher DON levels reflect much stronger secondary development of the pathogens in the batch—the pathogens have colonized the unturned malt much easier than they did the turned one (see Figure 2). Therefore, turning is a strong limiting factor preventing pathogen development and mycotoxin production. The relative content of DON-3G (in relation to DON) in the samples of malted grain not subjected to the aeration process was 43% (Legenda variety) and 21% (Pokusa variety), while the relative content of NIV3G was 68%. DON3G/DON and NIV3G/NIV ratios in malt made of both turned/aerated and unturned batches of the Legenda wheat variety were close to each other, but DON3G/DON ratio in the Pokusa wheat variety (21%) was lower and closer to the values in dry and steeped grain. That may suggest that the Pokusa variety was more contaminated with fungi mycelium capable to biosynthesise DON at a higher rate. Hampering of the DON detoxification reactions at some stage of life of the developing germs may also be an explanation.

## 3. Conclusions

The results indicate that the endogenic enzymes in germinating grains (responsible for plant detoxification) transform the main mycotoxins, DON and NIV, into their glucosides, DON3G and NIV3G, in the initial stages of the malting process. However, since the entire grain malting process is an opportunity for secondary development of the *Fusarium* pathogens (and consequently for biosynthesis of additional amounts of mycotoxins), the concentrations increased sharply on day 6 and 7 of the process: DON by 185–205% (depending on the wheat variety) on day 7 in comparison to day 2 (the steeped grain), NIV by 33%, DON3G by 321–548%, and NIV3G by 220%. Regular turning/aeration of the malted batch mechanically damages mycelial hyphae, which considerably hampers pathogen development and, consequently, the biosynthesis of additional amounts of mycotoxins. The transformation dynamics of DON, DON3G, and NIV during the wheat grain malting process have been studied before. However, the transformation dynamics of NIV3G are being reported for the first time in this study. Knowledge of mycotoxin metabolism during the grain malting process is invaluable from the perspective of food safety. Mechanisms controlling the usefulness of malted wheat in the food industry and the susceptibility of different wheat varieties to pathogen infections also require a deeper insight.

## 4. Materials and Methods

### 4.1. Chemicals and Reagents

Most of the certified standards used in this study were purchased from Romer Labs (Tulln, Austria). These include 100 µg/mL DON in acetonitrile, 50 µg/mL DON3G in acetonitrile, 50 µg/mL NIV in acetonitrile, ^13^C-labelled DON, and ^13^C-labelled NIV. NIV3G was extracted in-house from wheat, according to the method proposed by Yoshinari et al. [39]; the NIV3G concentration in the standard solution obtained was 110 μg/mL. All standard solutions were stored at 4 °C. Liquid chromatography–mass spectrometry (LC-MS) grade water and methanol were purchased from Witko (Łódź, Poland). Deionised water used to extract the analytes was produced in-house by a Hydrolab (Straszyn, Poland) demineraliser.

### 4.2. Samples

Two portions (1500 g each) were sampled from the grains of two winter wheat cultivars, Legenda and Pokusa, which were cultivated on test plots run by the National Research Institute of Plant Protection in Poznań, Poland, and harvested in 2018. The grains were inoculated with *F. culmorum* (0.5 × 106 spores per mL, strain KZF-5 from the collections of the Institute’s Research Centre for Registration of Agrochemicals). The inoculated grains were stored at 4 °C until further processing. Portions containing approximately 200 g of the grains were ground for 1 min in a Grindomix GM200 (Retsch, Haan, Germany) mill at 10,000 rpm. Two portions of air-dried wheat (each approximately 1.3 kg) were malted at the micromalting plant (Beer and Malt Laboratory, Prof. Waclaw Dabrowski Institute of Agricultural and Food Biotechnology, Warsaw, Poland) in compliance with the 1.5.3 MEBAK (Mitteleuropäische Brautechnische Analysenkommission) ed. 2011 guidelines, with some modifications in the degree of steeping and time of germination. A combination of steeping periods and air–rest periods was applied. The temperature of both water and air was 14 ± 1 °C, and the relative humidity was above 95%. The steeping schedule included 7 h of steeping, 17 h of air rest, 7 h of steeping, and 17 h of air rest again. After the steeping and air rests, the degree of steeping (determined by weight) was increased to 44%, by spraying. The steeped grain was split into two parts (of approximately 1 kg and 0.3 kg) and germinated for 7 days at 14 ± 1 °C. During the entire germination process, the grain in the 1 kg batch was turned and aerated once a day, and a sample of approximately 100 g was collected each day. The other batch, of 0.3 kg, was left undisturbed during the malting process. The moisture content in each collected sample was determined in compliance with the Polish standards PN-A-79083-5:1998 and PN-R74110:1998 (weighted dry mass method). All samples were frozen below −30 °C until analysis.

### 4.3. Sample Preparation

Before analysis, the samples were placed in dry ice. The frozen material was ground in a laboratory mill similar to the wheat grain. The LC-MS method (the “dilute-and-shoot” approach) was used to analyse the mycotoxins. In a 50 mL flask, 0.50 ± 0.05 g of the ground material and 10 µL each of ^13^C-labelled DON and NIV internal standards were mixed, and extracted with 2 mL of 8:2 (*v*/*v*) methanol:water mixture. Each sample was homogenised in a Unidrive X1000 homogeniser (CAT Scientific Inc., Paso Robles, CA, USA) at 10,000 rpm for 3 min. Further, the samples were centrifuged in an MPW 351R centrifuge (Med. Instruments, Warsaw, Poland) at 10,000 rpm for 10 min. The supernatant was filtered through a 0.45 μm mesh syringe filter. Further, 0.4 mL of the filtrate was mixed with 0.6 mL of water and filtered through a 0.2 μm mesh syringe filter. The final filtrate was subjected to LC-MS analysis.

### 4.4. UPLC-MS Analysis

An H-class ultra-high-performance liquid chromatograph (UPLC; Waters Corporation, Milford, MA, USA) coupled with a high-resolution time-of-flight mass spectrometer (TOF-HRMS) was used. The analytes were separated using a Cortecs C18 UPLC chromatographic column (2.1 mm × 100 mm, 1.6 μm; Waters, Milford, MA, USA) equipped with a suitable pre-column, both working with a gradient. The mobile phases were a 90:10 (*v*/*v*) methanol:water mixture (phase A) and 10:90 (*v*/*v*) methanol:water mixture (phase B). Ammonium formate (5 mM) and formic acid (0.2%) were added to both phases. The flow rate was 0.3 mL/min. The applied gradient was 100% B between 0 and 2 min, 50% B between 3 and 6 min, 100% A between 8 and 13 min, and 100% B between 14 and 18 min. Five microlitre volumes of the mobile phase were injected into the column. The spectrometer was operated in ESI ionisation mode at negative polarity. The ion source and desolvation temperatures were 120 °C and 230 °C, respectively. The nitrogen and drying gas flow rates were 650 L/min and 20 L/min, respectively. The capillary bias was 2400 V. The ion optics worked in the V mode. The spectrometer was calibrated using leucine-enkephalin.

### 4.5. Method Validation

The linearity range, limit of detection (LOD, analyte concentration at which signal:noise = 3:1), limit of quantification (LOQ, analyte concentration at which signal:noise = 10:1), recovery rate, and method repeatability (expressed as relative standard deviation, RSD) were determined for each analyte to validate the analytical method developed in this study. To eliminate matrix effects that could suppress analyte ions, calibration curves were obtained using blank solutions of wheat and its green malt, with added internal ^13^C-labelled standards at nine concentrations spanning the following ranges: 83–2000 μg/kg for NIV, 33–792 μg/kg for NIV3G, 167–20,000 μg/kg for DON, and 83–6000 μg/kg for DON3G. The coefficient of determination (R^2^) exceeded 0.99 in all cases. If the concentration of an analyte in a sample exceeded the method’s upper limit of linearity for that analyte, the sample was diluted and re-analysed. Recovery rates (R) were determined on the basis of analyses of samples to which known amounts of analytes were added at four fortification levels (apart from the internal standards). These samples were processed in the same manner as the unknowns (Section 4.3). The analysed ions, retention times, LOD, and LOQ for individual analytes are shown in Table 3. Further, the recovery rate (R%) and method repeatability (RSD%) are shown in Table 4.

The European Commission has established some criteria for analytical methods for various mycotoxins, including DON. The regulation No. 401/2006 of 23 February 2006 [40] lays down methods of sampling and analysis for the official control of mycotoxin levels in foodstuffs. For concentrations of mycotoxins ranging from 100–500 µg/kg, the regulation requires DON recovery rates within 60–110%. For concentrations greater than 500 µg/kg, the DON recovery rates should be within 70–120%. RSD, which reflects the method’s precision, must not be higher than 20% for both concentration ranges. No criteria have been set in the regulation for the remaining analytes investigated in this study. However, they all are trichothecenes of a similar chemical structure, so we adopted the same criteria for them, as specified for DON. The method developed in this study meets these criteria (see Table 4).

### 4.6. Statistical Analysis of the Experimental Data

The results were statistically analysed using the Statgraphics 4.1 (StatPoint Technologies, Inc., Warrenton, VA, USA) software package. One-way analysis of variance (ANOVA) was performed at a significance level of α = 0.05. Homogenous groups were determined using Fisher’s least significant difference (LSD) test.

## Figures and Tables

**Table 1:** Concentration of mycotoxins (µg/kg) in samples collected at successive stages of the wheat grain malting process. Homogenous groups of results have been marked with identical letters.

Wheat Variety	Mycotoxin	Concentration ± SD (µg/kg)								
		I	II	III	IV	V	VI	VII	VIII	IX
		Dry Grain	Grain after Steeping	Day 2	Day 3	Day 4	Day 5	Day 6	Day 7	Day 7 (Unturned Batch)
Legenda	DON	^a^ 1540 ± 332	^a^ 986 ± 103	^a^ 1178 ± 98	^a^ 939 ± 105	^a^ 1190 ± 238	^a^ 1349 ± 122	^b^ 2349 ± 267	^b^ 3010 ± 338	^c^ 7077 ± 1006
	DON3G	^a^ 236 ± 12	^a^ 285 ± 15	^a^ 428 ± 46	^b^ 467 ± 17	^c^ 907 ± 222	^c^ 1146 ± 191	^d^ 1744 ± 191	^d^ 1850 ± 397	^e^ 4751 ± 569
	NIV	^d^ 702 ± 64	^c^ 519 ± 6	^c^ 453 ± 10	^ab^ 328 ± 59	^ab^ 314 ± 34	^a^ 270 ± 24	^bc^ 411 ± 39	^d^ 691 ± 65	^e^ 841 ± 62
	NIV3G	^b^ 239 ± 45	^ab^ 220 ± 27	^a^ 163 ± 15	^ab^ 169 ± 11	^c^ 365 ± 90	^c^ 413 ± 27	^c^ 426 ± 44	^d^ 705 ± 13	^e^ 881 ± 54
Pokusa	DON	^bc^ 2164 ± 210	^ab^ 1642 ± 48	^a^ 1239 ± 178	^a^ 1145 ± 106	^ab^ 1216 ± 119	^c^ 2770 ± 375	^c^ 2953 ± 135	^d^ 4678 ± 963	^e^ 20,444 ± 873
	DON3G	^a^ 584 ± 143	^a^ 620 ± 87	^a^ 773 ± 113	^a^ 710 ± 97	^a^ 938 ± 101	^b^ 1533 ± 229	^c^ 2050 ± 144	^d^ 2609 ± 477	^e^ 6672 ± 667

a, b, c, d, e—Different letters describe significant differences (α = 0.05).

**Table 2:** Change (%) in mycotoxin concentration between selected stages of the wheat malting process. See Table 1 for stage designations.

Wheat Variety	Mycotoxin	II/I	III/II	IV/III	V/IV	VI/V	VII/VI	VIII/VII	VIII/II	IX/II	VIII/I	IX/I	IX/VIII
Legenda	DON	−36	+19	–20	+27	+13	+74 *	+28	+205 *	+617 *	+95 *	+360 *	+135 *
	DON3G	+21	+50	+9	+94 *	+26	+52 *	+6	+548 *	+1565 *	+683 *	+1913 *	+157 *
	NIV	–26 *	–13	–28 *	–4	–14	+52 *	+68 *	+33 *	+62 *	−2	+20 *	+22 *
	NIV3G	–8	–26	+3	+116 *	+13	+3	+65 *	+220 *	+300 *	+196 *	+270 *	+25 *
Pokusa	DON	–24	–25	–8	+6	+128 *	+7	+58 *	+185 *	+1145 *	+116 *	+845 *	+337 *
	DON3G	+6	+25	–8	+32	+63 *	+34	+27	+321 *	+976 *	+347 *	+1042 *	+156 *

* Statistically significant changes (α = 0.05).

**Table 3:** The analysed ions, retention times, LOD, and LOQ for individual analytes.

Analyte	Ion Mass (*m*/*z*)	Retention Time (min)	LOD (μg/kg)	LOQ (μg/kg)
DON	341.2 (M+FA-H)^−^	4.19	167	557
DON3G	503.2 (M+FA-H)^−^	4.33	83	277
NIV	357.2 (M+FA-H)^−^	2.30	83	277
NIV3G	519.2 (M+FA-H)^−^	2.42	33	110

**Table 4:** Recovery rate (R%) and method repeatability (expressed as relative standard deviation, RSD%) for individual analytes at four different fortification levels.

Recovery Rate (R) and Relative Standard Deviation (RSD)	DON	DON3G	NIV	NIV3G
fortification level (*n* = 4)	1200	400	125	50
R (%)	97.8	82.3	90.6	105.8
RSD (%)	18.3	10.1	14.0	13.4
fortification level (*n* = 4)	5000	1500	500	198
R (%)	92.2	86.0	92.3	88.0
RSD (%)	13.1	10.1	9.2	11.3
fortification level (*n* = 4)	10,000	3000	1000	396
R (%)	103.3	98.3	97.1	95.4
RSD (%)	5.4	6.6	9.4	11.3
fortification level (*n* = 4)	20,000	6000	2000	792
R (%)	91.1	84.4	93.3	86.3
RSD (%)	4.1	9.8	9.2	11.6

## Data Availability

Not applicable.
